# Devastating Transboundary Impacts of Sea Star Wasting Disease on Subtidal Asteroids

**DOI:** 10.1371/journal.pone.0163190

**Published:** 2016-10-26

**Authors:** Diego Montecino-Latorre, Morgan E. Eisenlord, Margaret Turner, Reyn Yoshioka, C. Drew Harvell, Christy V. Pattengill-Semmens, Janna D. Nichols, Joseph K. Gaydos

**Affiliations:** 1 One Health Institute, School of Veterinary Medicine, University of California Davis, Davis, California, United States of America; 2 Department of Ecology and Evolutionary Biology, Cornell University, Ithaca, New York, United States of America; 3 Marine Science Center, Northeastern University, Nahant, Massachusetts, United States of America; 4 Reef Environmental Education Foundation (REEF), Key Largo, Florida, United States of America; 5 The SeaDoc Society, Karen C. Drayer Wildlife Health Center - Orcas Island Office, University of California Davis, Eastsound, Washington, United States of America; Evergreen State College, UNITED STATES

## Abstract

Sea star wasting disease devastated intertidal sea star populations from Mexico to Alaska between 2013–15, but little detail is known about its impacts to subtidal species. We assessed the impacts of sea star wasting disease in the Salish Sea, a Canadian / United States transboundary marine ecosystem, and world-wide hotspot for temperate asteroid species diversity with a high degree of endemism. We analyzed roving diver survey data for the three most common subtidal sea star species collected by trained volunteer scuba divers between 2006–15 in 5 basins and on the outer coast of Washington, as well as scientific strip transect data for 11 common subtidal asteroid taxa collected by scientific divers in the San Juan Islands during the spring/summer of 2014 and 2015. Our findings highlight differential susceptibility and impact of sea star wasting disease among asteroid species populations and lack of differences between basins or on Washington’s outer coast. Specifically, severe depletion of sunflower sea stars (*Pycnopodia helianthoides*) in the Salish Sea support reports of major declines in this species from California to Alaska, raising concern for the conservation of this ecologically important subtidal predator.

## Introduction

Although not as well recognized or well documented, infectious disease can be as important as predation in structuring ecological communities [1,2,3]. Compared to terrestrial communities, however, far less is known about the distribution and spread of marine diseases where the open nature of marine ecosystems creates the potential for faster spread than on land [4]. Recent examples of widespread marine diseases with large community impacts include the near extirpation of a top predator, the intertidal sea star *Heliaster kubiniji* from Gulf of California, due to a devastating epidemic, and resulting expansion of a rock snail competitor, *Morula ferruginosa* [5], the mass mortality of the urchin *Diadema antillarum* from Caribbean reefs and subsequent macroalgal cover of corals [6,7], and the withering syndrome-related extirpation of black abalone (*Haliotis cracherodii*) from California reefs and the sequential increases of sessile invertebrates, algae and sea urchins [8].

The massive 2013–14 asteroid mortality event, sea star wasting disease (SSWD), extended from Mexico to Alaska devastating over 20 species of asteroids [9] and is currently the largest marine epizootic of a non-commercial marine taxon [9,10]. Sea star mortalities during this event were linked to a sea star-associated densovirus (SSaDV, family *Parvoviridae*) based on evidence provided by experimental challenge studies and a metagenomic analysis of field samples [9]. Infected sea stars (hereafter ‘stars’) develop lesions in the dermis that increase in depth and diameter, dissolving tissue from the outside in [9,10]. One or all arms then detach from the central disc as individuals die, often leaving only white piles of ossicles and disconnected limbs. Previous outbreaks of SSWD have been reported in the northeast Pacific, California and northern New England since the 1970s [11–13]; however, these earlier SSWD outbreaks involved single species in localized areas. In addition to the SSaDV, the 2013–14 SSWD epidemic has been linked to an increase [10,13,14], or decrease [15] in sea temperature, while host immunity [16], genetics [17], and a suite of other host or environmental factors are likely involved.

In temperate marine habitats less is known about the impacts and ecological effects of infectious disease in subtidal compared to intertidal communities. For example, 70% of reproductive adults of the intertidal ochre star (*Pisaster ochraceus*) were killed by SSWD in the San Juan Islands (SJI) of Washington State in the 2014–15 period [10] and 59–80% in Oregon [15], but data on the effect of SSWD on subtidal species across the entire range is lacking [10]. Despite the difficulty inherent in obtaining such data, this information is crucial to understand the current population status of subtidal star species and evaluate potential resulting community changes.

In the Salish Sea, a transboundary US/Canadian temperate marine ecosystem, anecdotal reports suggests that virtually all of the most common subtidal species were affected by SSWD, including: blood star (*Henricia spp*.), giant pink star (*Pisaster brevispinus*), leather star (*Dermasterias imbricata*), morning sun star (*Solaster dawsoni*), mottled star (*Evasterias troschelii*), ochre star (*Pisaster ochraceus*), rainbow star (*Orthasterias koehleri*), drab six-armed star (*Leptasterias hexactis*), Stimpson’s sun star (*Solaster stimpsoni*), sunflower star (*Pycnopodia helianthoides*) and vermillion star (*Mediaster spp*). Quantification of SSWD impacts in these species is lacking and understanding resulting community changes in this important ecosystem is critically needed.

We used two different dive survey methods to evaluate the species-specific effects of the SSWD outbreak in the Salish Sea. Long-term data on subtidal asteroid species, collected by roving diver surveys in five discrete oceanographic basins and on Washington State’s Outer Coast, were analyzed to evaluate potential species and basin-specific impacts of SSWD. Finer-scale, shorter-term sampling was conducted in the biodiverse SJI using strip-transect surveys to examine SSWD prevalence during the outbreak and species-specific changes in density.

## Materials and Methods

Animals were observed but not collected. No permits were required for this research.

### Study area

**Fig 1:**
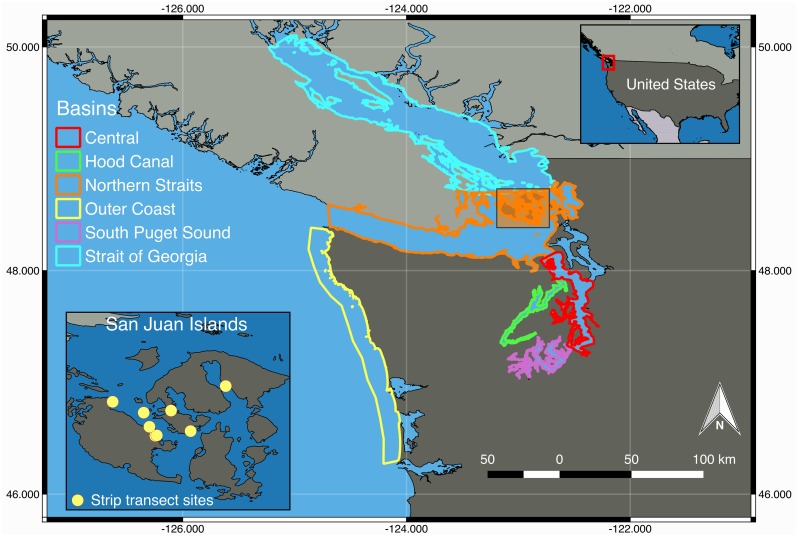
The 5 basins of the Salish Sea and the Outer Coast included in the study, and the sites in the San Juan Islands where the strip transects were completed.

We studied the potential impacts of SSWD in the Salish Sea, an inland marine ecosystem shared between British Columbia, Canada and Washington State, United States [18]. Based on subtidal sills and oceanic and freshwater flow patterns, oceanographers divide this 16,925 km^2^ transboundary ecosystem into 6 sub-basins: the Strait of Georgia, Northern Straits, Whidbey Basin, Central Basin, Hood Canal, and South Puget Sound [19,20]. Sufficient data existed for evaluating the subtidal impacts of SSWD in 5 of the 6 sub basins as well as on Washington State’s Outer Coast (hereafter ‘Outer Coast’; Fig 1).

### Subtidal Roving Diver REEF surveys

Data collected by trained and tested recreational scuba divers between January 1, 2006 and December 31, 2015 and submitted to the Reef Environmental Education Foundation (REEF) Volunteer Fish Survey Project Database [21] were analyzed. Using the Roving Diver Technique [22] surveyors record sightings of positively identified species and at the end of the dive, assign them to one of four abundance index categories: ‘Single’ (1 individual), ‘Few’ (2–10 individuals), ‘Many’ (11–100 individuals), and ‘Abundant’ (>100 individuals) [23]. Data used were restricted to the 3 asteroids and two urchin species for which data were available: *D*. *imbricata*, *P*. *brevispinus*, *P*. *helianthoides*, green sea urchin (*Strongylocentrotus droebachiensis*) and red sea urchin (*Mesocentrotus franciscanus*). To standardize survey habitat type to rocky substrate and minimize potential observational bias we used data from surveys conducted in habitats not classified as artificial reefs, sandy bottom, eelgrass beds and mud/silt bottoms and from dives conducted between 4am-10pm that lasted 20–80 minutes. *M*. *franciscanus* are harvested in the study area, therefore, we compared results for this species when using the complete dataset versus restricted to fishing closures within the study area [24] in order to evaluate potential bias caused by human harvesting.

We calculated the Abundance Score [23] for the 3 asteroids and both urchin species in each of the 5 isolated oceanographic basins and in the Outer Coast for every year between 2006–2015. We added a new category in the abundance index with value zero for those species not reported by a determined surveyor. The training required for surveyors and the ease of correctly identifying the asteroid species involved allowed us to safely assume actual absence. We used these absence records to calculate the Sighting Frequency (SF; the proportion of surveys each species was reported by basin per year), a necessary factor to estimate the Abundance Score (hereafter ‘abundance’) [23]. Moreover, in order to present a comprehensive numerical scale of the impact of SSWD on *P*. *helianthoides*, we used data collected by 12 expert-level REEF surveyors at 10 dive sites within the SJI in September 2013, for a total of 114 roving surveys. These divers, in addition to the data on abundance, recorded actual counts for this species. The median of the distributions of counts between 2–10 and 11–100 were used to estimate their actual number when abundance category ‘Few’ and ‘Many’ were recorded for this species. When abundance category ‘Abundant’ was assigned, we used the lower boundary of this category: 101 individuals. In this manner we transformed the non-linear scale to a better-comprehended linear scale. With these values we estimated the *P*. *helianthoides* mean count in isolated oceanographic basin per year.

### Strip transect surveys

Diving surveys were conducted by trained and tested scientific divers in March-August 2014–15 at eight sites in the San Juan Islands (Fig 1). Sites were geographically distinct, such that it was assumed that they represented discrete subtidal star populations. These sites included Bell Island (48.59549775, -122.9813218), FHL Dock (48.5454276, -123.0125132), Lab 11 Beach (48.54615651, -123.0096835), Reuben Tarte (48.61301143, -123.097432), Rosario (48.64422196, -122.8726172), Shaw House (48.55462263, -122.9424861), Strathmann's (48.56333938, -123.0242747), and Yellow Island (48.59136076, -123.035717). Surveys were repeated at least once in seven of the eight sites in spring/summer 2014, before the high SSWD mortality occurred, and again in spring/summer 2015, after SSWD peak prevalence. Several sites were surveyed multiple times during summer 2014, when our observations suggested that the outbreak was at peak prevalence in the survey area.

The entry point for each dive site was fixed using GPS coordinates and shore landmarks. Divers entered the water near shore and followed the rock wall down until reaching sloping bedrock habitats. In these zones a team of two divers laid a 20 or 25m transect at depths between 6 and 18m, depending on the landscape, and maintained depth along a single transect. Typically two transects were run at each site; however due to degree of current, depth, and number of personnel for each dive, the number of transects per survey ranged from one to seven. In general, transects were laid within a ~90m distance from the entry point and were separated by at least 5m. Transects start points did not correspond between surveys, but they were consistently laid in comparable habitat conditions.

Divers were allowed to move understory kelp to search both sides of the blades and the rock surfaces underneath, however rocks were left in place. Flashlights were used to check crevices and the underside of rocks. Each diver tallied the species and health of every star observed within 2m of either side of the transect tape. When reaching the end of the transect, the tape was rolled up and the divers continued swimming along the survey zone to begin the next transect, in the direction away from the previous transect so as to not overlap.

The health of the stars was visually determined for *E*. *troschelii*, *Henricia spp*. and *P*. *helianthoides*, without picking them up or disturbing them. The case definition for SSWD was: presence of white lesions of exposed tissue and/or recent arm loss. If in doubt, a star was scored as healthy. In the case of sick *P*. *helianthoides*, which would often lose multiple arms, many of which would continue traveling without the central disc, special care was taken not to double count stars due to roving severed arms. For each survey, divers conferred and agreed upon the number of *P*. *helianthoides* seen, taking into account the number of central discs found and the colors of the individual roving arms (i.e. blue vs orange star) seen. Prevalence of disease in spring/summer 2014 was estimated as the proportion of stars fulfilling the case definition. Moreover, the density of *D*. *imbricata*, *E*. *troschelii*, *Henricia spp*., *L*. *hexactis*, *Mediaster spp*., *O*. *koehleri*, *P*. *brevispinus*, *P*. *helianthoides*, *P*. *ochraceus*, *S*. *dawsoni* and *S*. *stimpsoni* was estimated during spring/summer 2014 and 2015.

### Statistical analysis

#### 1. Subtidal roving diver REEF surveys

The annual abundance of the pre-epidemic period (2006–13) for specific species and basins were used in the fitting process of autoregressive integrated moving average models: ARIMA(*p*,*d*,*q*) [25] where *p* indicates the autoregressive (AR) term, *d* the differencing order necessary to achieve stationarity and *q* is the moving average (MA) order. These models took into consideration the correlation of the abundance scores over time and depending only on their past values and random shocks. We used the autocorrelation function (ACF) and the partial autocorrelation function (PACF) to determine the initial AR and MA terms. We fitted models with varying AR and MA orders using R program version 3.2.2 [26] and the ‘forecast’ package [27]. To accomplish the parameter estimation, we selected the conditional-sum-of-squares method which first finds starting values and then performs maximum likelihood. Given the sample size (8 time points), stationarity of the series was evaluated based on the values of the estimated AR or MA coefficients (no hypothesis testing involved) and seasonality was not considered. Model selection was based on the maximum likelihood estimate of the residuals variance (σ^2^) and the corrected Akaike’s Information Criterion (AICc) [28] for its superiority over AIC [29]. For both indicators we targeted the lowest values. The residuals of the selected model were inspected for absence of autocorrelation and normal distribution. Parsimony of the ARIMA(*p*,*d*,*q*) models was prioritized; therefore, intercept models were selected when they implied the lowest σ^2^ and AICc. The resulting models were subsequently used to forecast the abundance scores in the epidemic period (2014–15) for each species and basin. In the case of intercept only models or ARMA(0,0), the forecasting values for these 2 years corresponded to the mean pre-epidemic abundance score. We considered a biologically meaningful change of the population due SSWD if the observed abundance in the epidemic period were outside of the 95% confidence interval (CI) of the ARIMA forecasted (hereafter ‘projected’) abundance for 2014–15.

#### 2. Strip transect surveys

All analyses were conducted in R [23]. We assessed differences in the likelihood of SSWD presence between *E*. *troschelii*, *Henricia spp*. and *P*. *helianthoides* among months for spring/summer 2014 (specifically May to August). We constructed a logistic regression model in which the outcome was the presence of SSWD in a given star (*y*_*i*_ = 0 if healthy and 1 otherwise, according to the case definition). Here, P(*y*_*i*_ = 1) = *p*_*i*_, where *p*_*i*_ is the probability of SSWD in the *i*^th^ star. This term was related to our fixed predictors of interest: the asteroid species and months.

$$logit(p_{i})\;=\;\alpha_{0}\;+\;\beta_{1}E_{i}+\beta_{2}P_{i}+\beta_{3}Jun_{i}+\beta_{4}Jul_{i}+\beta_{5}Aug_{i}$$

where *E*, *P*, *Jun*, *Jul* and *Aug* indicate *E*. *troschelii*, *P*. *helianthoides*, June, July and August, respectively for the *i*^th^ observation; therefore we used *Henricia sp*. and May as references for species and months. We assumed all star observations were independent within a single spatial unit, the SJI. Model fitting was achieved with maximum likelihood in the lme4 package [30] and the appropriateness of including both sets of categorical covariates (species and months) to improve model fitting was evaluated using the AIC. Posteriorly, we performed Bonferroni corrected contrasts (overall α = 0.05) to determine differences in log odds estimates between species and between months using the multcomp package [31].

We evaluated differential impacts of SSWD among *D*. *imbricata*, *E*. *troschelii*, *Henricia spp*., *L*. *hexactis*, *Mediaster spp*., *O*. *koehleri*, *P*. *brevispinus*, *P*. *helianthoides*, *P*. *ochraceus* and *S*. *stimpsoni* by estimating changes in their density during the epidemic period (between 2014–15). For this purpose we constructed a multilevel Bayesian *Poisson* regression using JAGS 4.1.0 [32] in R 3.2.2 [26] with R2jags 0.5–7 [33] as the interface (S2 Appendix). We assumed that conditional on the respective means λ_*ij*,_ the number of stars of the *j*^th^ taxa observed in the *i*^th^ survey followed a Poisson distribution: y_*ij*_ ~ *Poisson* (A_*ij*_λ_*ij*_). The A_*ij*_ was the area surveyed in the *i*^th^ survey for the *j*^th^ taxon corresponding to the ‘offset’. We modeled λ_*ij*_ via a log link function as:

$$log\left(\lambda_{ij}\right)=log\left(A_{ij}\right)\;+\;\alpha_{j\left[i\right]}\;+\;\beta_{j\left[i\right]}Year_{i}\;+{\;\varepsilon }_{ij}$$

where ε_*ij*_ allows for overdispersion [34], α_*j*_ and β_*j*_ are the specific *j*^th^ taxon intercept and slope, respectively; and *Year*_*i*_ is a categorical variable with *Year*_*i*_ = 0 if 2014, and 1 otherwise. Therefore, the α_*j*_’s are the estimated taxon log densities in 2014 while the β_*j*_’s are the taxon log density change in 2015. Here, ε_*ij*_ *~ N*(0, σ_ε_), with σ_ε_ expressing the amount of overdispersion in the data and

$$\left(\begin{array}{c} \alpha_{j}\\ \beta_{j} \end{array}\right)\sim \text{ N}\left(\left(\begin{array}{c} \mu_{\alpha }\\ \mu_{\beta } \end{array}\right),\;\left(\begin{array}{cc} \sigma_{\alpha }^{2} & {\rho \sigma }_{\alpha }\sigma_{\beta }\\ {\rho \sigma }_{\alpha }\sigma_{\beta } & \sigma_{\beta }^{2} \end{array}\right)\right)$$

for *j* = 1,…,10. In this model, μ_α_ and μ_β_ correspond to the mean of the α_*j*_’s and β_*j*_’s, respectively; σ_α_ σ_β_ are the corresponding standard deviations and ρ is the correlation between the α_*j*_’s and β_*j*_’s.

Non-informative priors or hyperpriors were assigned for μ_α_, μ_β_, σ_α_, σ_β_, σ_ε_ and ρ: Normal (0, 10,000) for the first two parameters, Uniform (0,100) for the third, fourth and fifth, and Uniform (-1,1) for the last one. Posterior parameter distributions were sampled from each of three chains for 60,000 iterations following a 10,000 iteration burn-in, and thinning set to 5, for a total of 30,000 samples. Each chain was assigned random start values; Normal (0,1) distribution for μ_α_, μ_β_ and the ε_*ij*_’s, and from a Uniform (0,1) for σ_α_, σ_β_, σ_ε_ and ρ. Convergence was assessed by the Gelman–Rubin statistic [35] and graphically. We considered a credible change in taxon density from 2014 to 2015 if the 95% credible interval of the corresponding *j*^th^ taxa slope, β_*j*,_ did not include zero.

## Results

### Subtidal roving diver survey data and ARIMA models

A total of 8,097 REEF surveys were analyzed. Insufficient data precluded evaluation of the Whidbey Basin. The total number of surveys per year in each of the 5 sub-basins included in the study and the Outer Coast were relatively consistent over the times analyzed (Table 1). Mean dive times were 46, 49, 54, 49, 51 and 52 minutes for the Strait of Georgia, Northern Straits, Central Basin, Hood Canal, South Puget Sound and the Outer Coast, respectively; with typical maximum dive depths between 12-21m. Most surveys were conducted between 0700–1900 hours and diver visibility ranged from 3—14m. Eight habitat types were represented: kelp forest, rock/shale reefs, open oceans, surf grass beds, pinnacles, bull kelp beds, cobblestone/boulder fields and walls, but most surveys were conducted in rock/shale reefs, walls and cobblestone/boulder fields. During the 8,097 dives, one or more of the 5 species analyzed were not sighted, necessitating us to include 20,264 absence records to complete the dataset.

**Table 1:** Number of REEF surveys conducted by year in each studied basin in the Salish Sea.

	Year									
Basin	2006	2007	2008	2009	2010	2011	2012	2013	2014	2015
Strait of Georgia	188	137	106	183	212	183	326	459	361	358
Northern Straits	182	213	227	303	330	198	258	322	302	309
Central Basin	46	62	98	113	136	84	95	104	96	144
Hood Canal	123	155	96	132	256	187	212	100	140	87
South Puget Sound	27	38	43	45	67	42	43	39	37	21
WA Outer Coast	0	6	8	12	11	10	10	12	2	1

**Fig 2:**
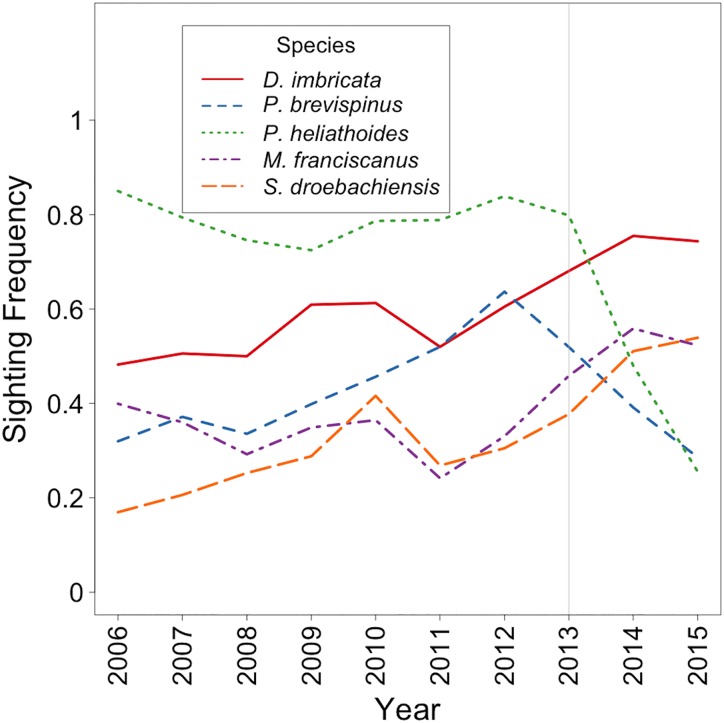
Sighting Frequency of *D*. *imbricata*, *P*. *brevispinus*, *P*. *helianthoides*, *S*. *droebachiensis* and *M*. *franciscanus* in 5 basins of the Salish Sea and the Outer Coast 2006–15. Grey line marks the epidemic onset.

Compared to pre-epidemic (2006–13), the SF of *P*. *helianthoides* declined markedly after onset of the epidemic (2014–15), while SF of *D*. *imbricata* and urchin species *S*. *droebachiensis* and *M*. *franciscanus*, trended upward throughout the entire time series (2006–2015; Fig 2). The SF for *P*. *brevispinus* began to decline in 2012 and continued to decline through 2015; however, the lowest SF during the decline (in 2015) was similar to the lowest SF between 2006–2011.

**Fig 3:**
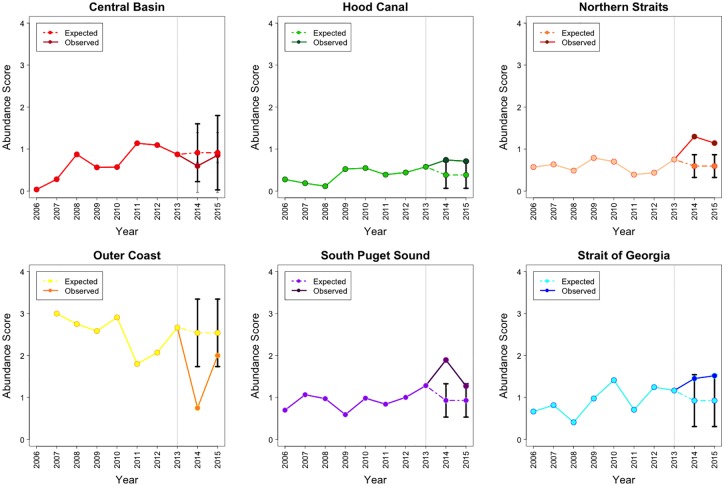
The actual 2006–15 and projected 2014–15 abundance for *D*. *imbricata* in 5 basins of the Salish Sea and the Outer Coast. Grey line marks the epidemic onset. Note: Abundance = Density Score x SF, where Density Score = [(nSx1)+(nFx2)+(nMx3)+(nAx4)] / (nS + nF + nM + nA). Here nS, nF, nM, and nA represent the number of times each abundance category was assigned for a given species.

Species and basin specific ARIMA models and their estimated parameter values are available in S1 Appendix. After the SSWD epidemic onset (in 2013), *D*. *imbricata* abundance increased in 3 basins (Hood Canal, the Northern Straits and the Strait of Georgia). Specifically, in the Northern Straits, *D*. *imbricata* abundance increased 1.51 times by 2015, exceeding the projection for the population trajectory. Although they did not exceed the population projected in the Hood Canal and the Strait of Georgia, the abundance of this species increased 1.23 and 1.3 times, by 2015, respectively. In the South Puget Sound basin, abundance increased above projected levels in 2014, then returned to within expected trends in 2015. There were no major changes for this species in the Central Basin, while the Outer Coast had a decreasing abundance trend, although the complete 10-year abundance trend in this basin was unstable when compared to the others (Fig 3).

**Fig 4:**
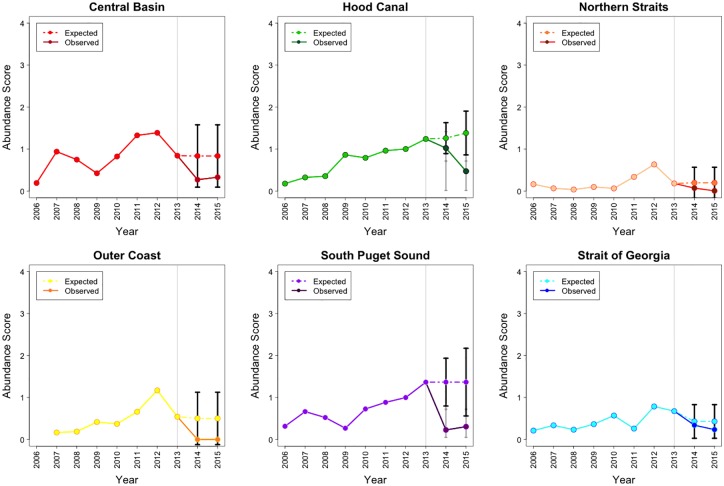
The actual 2006–15 and projected 2014–15 abundance for *P*. *brevispinus* in 5 basins of the Salish Sea and the Outer Coast. Grey line marks the epidemic onset. Note: Abundance = Density Score x SF, where Density Score = [(nSx1)+(nFx2)+(nMx3)+(nAx4)] / (nS + nF + nM + nA). Here nS, nF, nM, and nA represent the number of times each abundance category was assigned for a given species.

While historically *P*. *brevispinus* has been rarer than the other two star species monitored on roving dive surveys, abundance after 2013 did show a clear decline in all basins and on the Outer Coast, although the decline in abundance was beyond the 95% CI of projected values only in the Hood Canal and South Puget Sound basin. In the Central Basin, Northern Straits basin, Outer Coast and the Strait of Georgia basin, this downward trend started in 2012. By 2015, the abundance in these 3 Salish Sea basins corresponded to 0.39, 0.03 and 0.35 times the 2013 abundance respectively. On the Outer Coast the abundance reached 0 during 2015. Declines in abundance limited to the post-epidemic onset (2014–15) occurred only in the Hood Canal and South Puget Sound basins where the 2015 abundance corresponded to 0.38 and 0.22 times the 2013 abundance. In these 2 basins, the observed abundances post epidemic onset were not lower than the historical pre-epidemic estimated mean (Fig 4).

**Fig 5:**
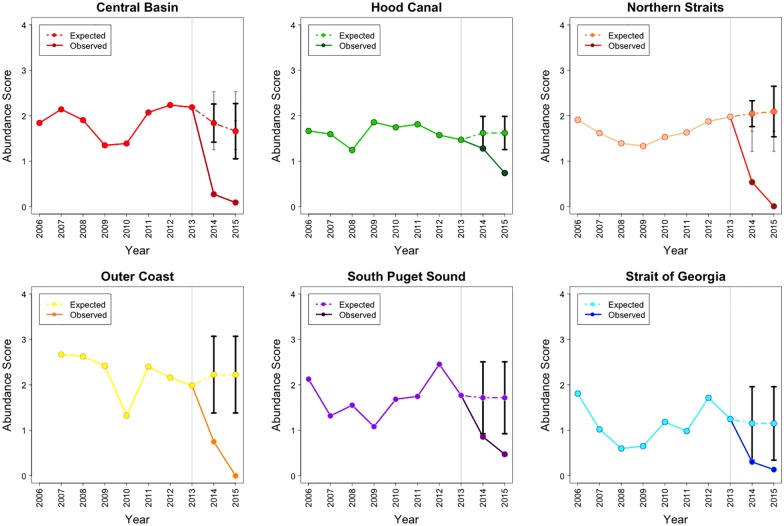
The actual 2006–15 and projected 2014–15 abundance for *P*. *helianthoides* in 5 basins of the Salish Sea and the Outer Coast. Grey line marks the epidemic onset. Note: Abundance = Density Score x SF where Density Score = [(nSx1)+(nFx2)+(nMx3)+(nAx4)] / (nS + nF + nM + nA). Here nS, nF, nM, and nA represent the number of times each abundance category was assigned for a given species.

**Fig 6:**
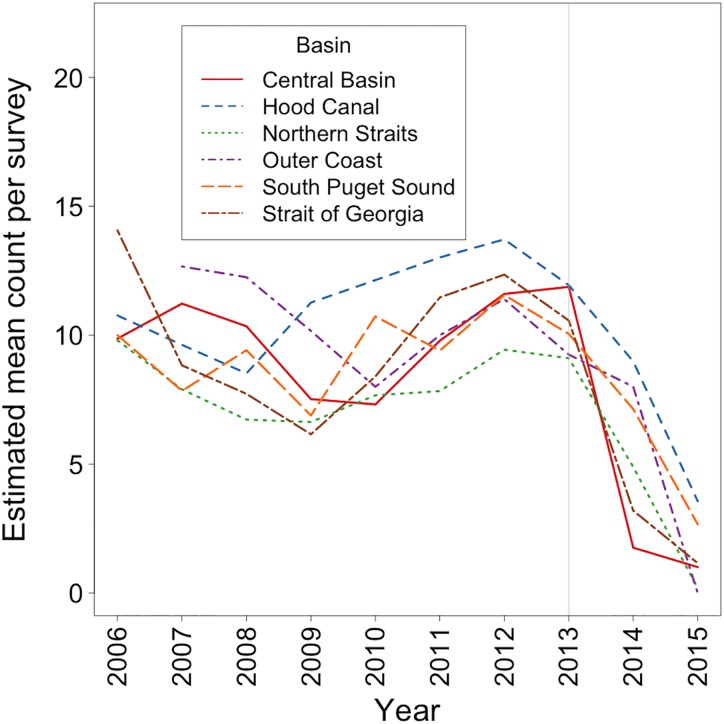
The 2006–15 estimated count of *P*. *helianthoides* in 5 basins of the Salish Sea and the Outer Coast. Grey line marks the epidemic onset.

Post epidemic onset, *P*. *helianthoides* declined markedly in every basin compared to the projected abundance (Fig 5). The most dramatic *P*. *helianthoides* declines were seen in the Central Basin, Northern Straits and the Outer Coast, where the abundance had been stable pre-epidemic onset, while in 2015 the observed abundances in these basins corresponded to 0.04, 0.005 and 0 times the 2013 value respectively. In the Hood Canal basin the decline was less severe (2015 abundance was 0.5 times the 2013 abundance) and the declining trend started earlier than 2013. In the South Puget Sound basin and the Strait of Georgia basin *P*. *helianthoides* abundance began to decline prior to 2013, but the decline dramatically increased between 2013 and 2015 (Fig 5). In 2015, the abundances in these basins corresponded to 0.27 and 0.11 times the 2013 abundance. Similarly, declines occurred in the Outer Coast reaching 0 abundance at the end of the study period. Estimated mean count for *P*. *helianthoides* per roving dive survey were consistent until 2013, averaging 9.89 individuals. In the Hood Canal basin, the Outer Coast, the South Puget Sound basin and the Strait of Georgia basin, the decline started in 2012 but decreased steeply during the post-epidemic period. In the Central Basin, Hood Canal basin, Northern Straits basin, the Outer Coast, South Puget Sound basin and the Strait of Georgia basin the mean count per dive decreased at rates -9.25, -8.91, -8.37, -9.41, -7.38 and -10.86, respectively between 2013–15. By 2015 there were zero or close to zero individuals per roving dive survey in the Central Basin, the Northern Straits basin, the Outer Coast and Strait of Georgia basin (Fig 6).

**Fig 7:**
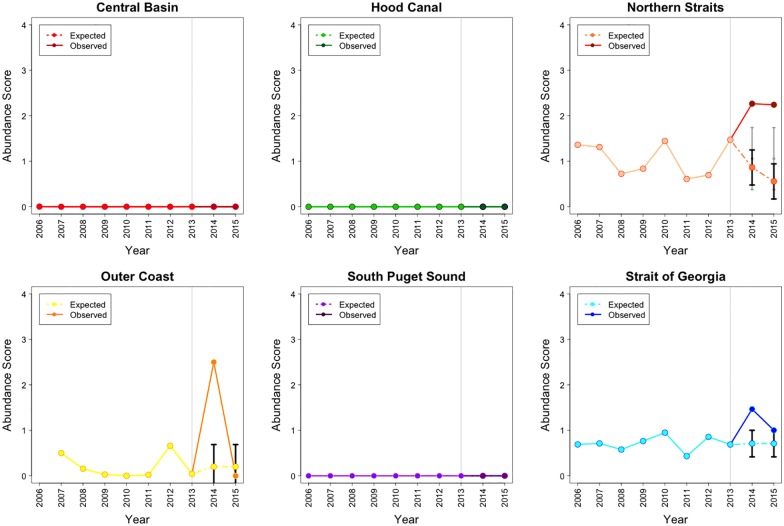
The actual 2006–15 and projected 2014–15 abundance for *M*. *franciscanus* in 5 basins of the Salish Sea and the Outer Coast. Grey line marks the epidemic onset. Note: Abundance = Density Score x SF, where Density Score = [(nSx1)+(nFx2)+(nMx3)+(nAx4)] / (nS + nF + nM + nA). Here nS, nF, nM, and nA represent the number of times each abundance category was assigned for a given species.

**Fig 8:**
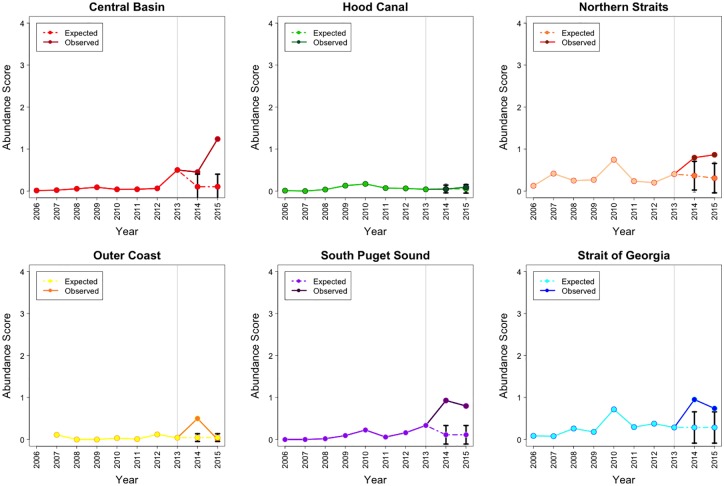
The actual 2006–15 and projected 2014–15 abundance for *S*. *droebachiensis* in 5 basins of the Salish Sea and the Outer Coast. Grey line marks the epidemic onset. Note: Abundance = Density Score x SF, where Density Score = [(nSx1)+(nFx2)+(nMx3)+(nAx4)] / (nS + nF + nM + nA). Here nS, nF, nM, and nA represent the number of times each abundance category was assigned for a given species.

By 2014, *M*. *franciscanus* abundance had increased in places where it was originally present (the Northern Straits basin, the Outer Coast and the Strait of Georgia basin), but in 2015 this trend continued beyond the CI of the projected abundance only in the Northern Straits (Fig 7). During the 2013–15 period *M*. *franciscanus* abundance in this basin increased 1.52 times. As urchins are harvested by tribal fisheries in Washington’s half of the Salish Sea, we compared increases in red urchin abundance with data taken only from dive sites located within urchin harvest closure zones that are present in the Northern Straits basin [21]. We did not find meaningful differences between abundances for this species in harvested versus fishery closure areas. After 2013, *S*. *droebachiensis* abundance clearly increased in the Central Basin, the Northern Straits basin, the South Puget Sound basin and the Strait of Georgia basin beyond projections. In these basins the abundances increased 2.47, 2.13, 2.38 and 2.58 times, respectively. Remarkably, in the Central Basin and South Puget Sound basin this species was historically rare, but became more abundant in 2013, with abundance increasing at even higher rates thereafter (Fig 8).

### Strip transect surveys

While reports of SSWD began in June and September 2013 on the Outer Coast and in the Strait of Georgia [9, 36], signs of widespread disease did not appear in the SJI until *P*. *helianthoides* being held in captive in tanks with open water systems began to decline in April 2014 according to our observations (MEE, CDH). In SJI strip transect surveys, initial SSWD signs appeared in *P*. *helianthoides* in May 2014. Disease prevalence increased through the summer of 2014 in the 3 asteroid taxa studied, but prevalence differed markedly by species, reaching ~50% for *P*. *helianthoides* and *E*. *troschelii* in August, while the maximum prevalence for *Henricia spp*. never exceeded ~5%. Interestingly, in July the prevalence of *E*. *troschelii* was low and similar to *Henricia spp*. (3%) but increased to approximate *P*. *helianthoides* prevalence in August (in July, *P*. *helianthoides* prevalence was 36%). The selected logistic regression included the species and months as associated with the presence of SSWD (Table 2; AIC = 375.788). The likelihood of SSWD was significantly higher for *E*. *troschelii* and *P*. *helianthoides* compared to *Henricia spp*., while the contrast between *E*. *troschelii* and *P*. *helianthoides* showed that SSWD was 3.12 times more likely in *P*. *helianthoides* (p-value = 0.02). Similarly, the likelihood of SSWD was markedly different among months with an increasing trend from May to August.

**Table 2:** Parameter estimates of the logistic regression for SSWD prevalence in 3 asteroid species during May—August 2014.

Parameter	Level	Estimate	Odds Ratio (95% CI)	S. E.	z-value	p-value
Intercept	-	-6.876	0.0001 (0.00022–0.0037)	0.715	-9.619	< 0.001
Species	*Henricia sp*.	0	1	-	-	
	*E*. *troschelii*	2.329	10.28 (3.86–29.15)	0.509	4.573	< 0.001
	*P*. *helianthoides*	3.484	32.59 (12.67–98.68)	0.520	6.696	< 0.001
Month	May	0	1	-	-	
	June	1.508	4.52 (1.66–15.81)	0.556	2.695	0.007
	July	2.642	14.04 (5.17–49.32)	0.562	4.706	< 0.001
	August	4.056	57.72 (16.11–254.33)	0.694	5.843	< 0.001

The total area surveyed was 2,160 and 2,080m^2^ in 2014 and 2015, respectively. Recorded densities of the different taxa varied differentially between 2014 and 2015. The density of the more common taxa, including *P*. *helianthoides*, *E*. *troschelii* and *Henricia spp*. consistently decreased from 5.5 to 0.18, 2.82 to 0.78 and 7.42 to 5.32 individuals per 100m^2^, respectively. Among less common subtidal taxa, density declined in *P*. *ochraceus* from 0.3 to 0.05 individuals per 100m^2^, while *P*. *brevispinus*, *O*. *koehleri* and *S*. *stimpsoni* declined from 0.25, 0.25 and 0.34 individuals per 100m^2^, respectively, to completely absence in 2015. However, density increased for *L*. *hexactis* and *Mediaster spp*. from being absent in 2014 to 0.05 and 0.15 individuals per 100m^2^ in 2015, respectively; while *D*. *imbricata* increased from 0.03 to 0.22 individuals per 100m^2^ after the epidemic onset. Finally, *S*. *dawsoni* was absent in both years, suggesting that this species, historically rare, could have been affected by SSWD prior to 2014 and reduced to an abundance below the detection threshold of the strip transects.

**Fig 9:**
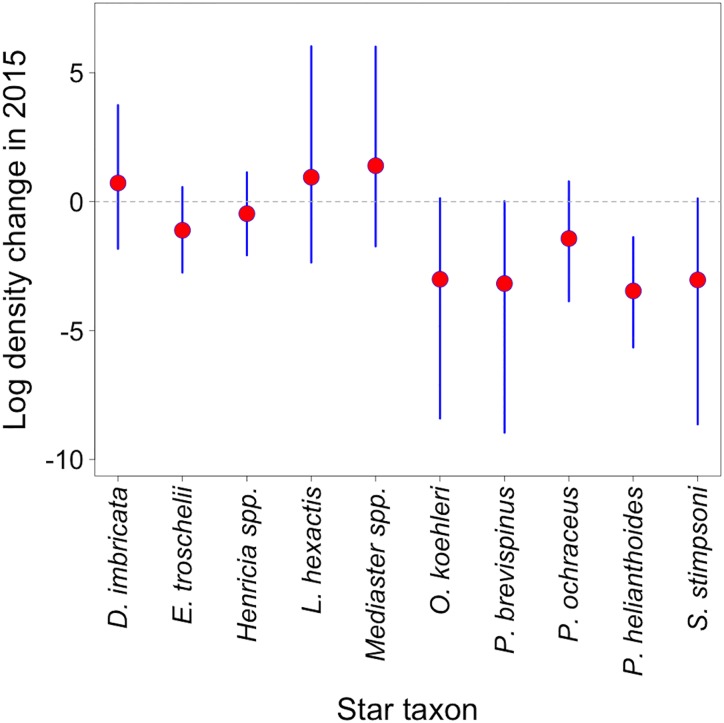
Estimated mean log density change in 2015 (the red point) for 10 asteroids and their corresponding 95% credible intervals (blue lines).

In the Bayesian multilevel Poisson regression, all Gelman-Rubin statistics were lower than 1.04, and graphics supported convergence of the chains. The effective sample size for all parameters was at least 240. Mean values for μ_α_ and μ_β_ were -6.561 and -1.258 indicating a general decrease in star density in 2015. As expected, there was an important difference in intercepts and slopes across taxon with mean σ_α_, and mean σ_β_, equal to 2.893 and 2.801, respectively. Finally, the mean ρ value was -0.384 indicating a negative association between intercepts and slopes. Model results showed that among common species, *P*. *helianthoides* had a credible decline between 2014–15. Uncommon species, *O*. *koehleri*, *P*. *brevispinus*, and *S*. *stimpsoni* had a decline in 2015 that was borderline credible; *D*. *imbricata*, *L*. *hexactis* and *Mediaster spp*. presented a non-credible increase in 2015; and *E*. *troschelii*, *Henricia spp*. and *P*. *ochraceus* had a non-credible decrease in 2015 (Fig 9).

## Discussion

### Epidemic onset and disease prevalence

Detecting wildlife epidemics is often challenging due to the difficulty of diagnosing disease in wildlife and the action of ecological processes that hide mortality such as predation of weak animals, scavenging of carcasses and decomposition [37–39]. Detection of marine epidemics is even more challenging than those occurring in terrestrial habitats due to the additional complexities of making field observations [4, 40]. The 2013–14 sea star wasting epizootic was detected due to the extreme lesions, arm loss and massive mortality occurring in 20 of the most common intertidal and subtidal asteroid species across a vast geographic range.

The exact onset of SSWD in the Salish Sea is unknown. Clinical signs and mortality associated with this disease were reported from the Outer Coast as early as June 2013 [9] and in the Strait of Georgia in September 2013 [36]. Over 100 subtidal roving diver surveys conducted in the Northern Straits basin in October 2013 detected a very small number of animals with lesions (specifically in individuals of *D*. *imbricata*, *E*. *troschelii*, *P*. *helianthoides*, and *S*. *stimpsoni*) from which SSWD-associated Densovirus was detected [9]. Anecdotal reports suggest the SSWD epidemic was observable in other Salish Sea basins and the Outer Coast during late 2013 and early 2014.

The present data show the pattern of emergence after 2013. Divers did not notice large-scale clinical signs and mortality until May 2014, when clinical disease was seen in free-ranging *P*. *helianthoides* present in the SJI strip transects. During the next month, sick *E*. *troschelii*, and *Henricia spp*. individuals were also observed. For all three species, prevalence rapidly increased to a peak in August, reaching ~ 50% in *P*. *helianthoides*, and around 47 and 5% prevalence in *E*. *troschelii* and *Henricia spp*., respectively.

### Species declines and species-specific differences

Data from strip transects and long-term data from roving diver surveys confirm that multiple species of asteroids have been affected by SSWD and significant species-specific differences exist. *P*. *helianthoides* was the species most dramatically impacted, showing the greatest rate of decline across all basins in the long-term data and locally in the SJI strip transect data. The estimated mean number of *P*. *helianthoides* counted per roving diver survey in the region showed a major shift, from 9.9 individuals in the pre-epidemic period to less than 2 individuals in 2015 after the emergence of SSWD.

The density of other common star species *E*. *troschelii* and *Henricia spp*., decreased in the post epidemic onset in the strip transects, but not credibly in the multilevel Poisson regression. The 2015 mean log density changes for these taxa were very close to zero.

Among less common stars, *O*. *koehleri*, *P*. *brevispinus* and *S*. *stimpsoni* were detected at low levels during 2014 strip transects, and were all but absent in 2015, suggesting that their populations had been reduced to densities at below detection limits in the strip transect study and seem to have been severely affected. These reductions in density were not statistically credible due to the low initial numbers, but we are confident that they are biologically relevant, because most of the credible interval is below zero. Roving diver surveys reveal that *P*. *brevispinus* declined in all basins and on the Outer Coast after 2013. While these declines were not beyond the CI of the projected abundances for the post epidemic period, they were steeper in the Central Basin, Hood Canal basin and the South Puget Sound basin as well as on the Outer Coast. The observed post epidemic abundance contained by the projected CI is likely the result of the historical lower abundance of this species. In 3 basins (Central Basin, Northern Straits and Strait of Georgia) and on the Outer Coast, *P*. *brevispinus* declines actually started in 2013. Without SSWD prevalence data pinpointing the level of wasting, it is challenging to assign cause, but these 2013 declines are likely due to SSWD as it was detected and shown to cause disease in other basins and species at that time [36,41].

*Pisaster ochraceus* is primarily an intertidal star with low subtidal density, but its disappearance from year 2 of the subtidal strip transects coincided with its major intertidal decline in the SJI and lower Puget Sound [10] as well as on the Oregon coast [15].

Even though SSWD has been previously documented in the wild as well as in captivity for other less common stars such as *D*. *imbricata*, *L*. *hexactis* and *Mediaster spp*., these species did not decline in the Salish Sea. The abundance of *D*. *imbricata* actually increased after 2013 in 3 basins. At the end of the study period, observed abundance in all 5 basins remained within the projected range except for the Northern Straits basin, where it was exceeded. This fact, combined with density increases in the strip transects in the SJI between 2014–15, suggests that this species is more resistant to SSWD than other stars. The densities of *L*. *hexactis* and *Mediaster spp*. on strip transects also tended to increase between 2014–15. However, they were absent in 2014 and were found in 2015.

In summary, two years of post epidemic onset data suggest that the largest impacts of SSWD occurred in *P*. *brevispinus*, *P*. *helianthoides*, *O*. *koehleri*, *S*. *dawsoni*, and *S*. *stimpsoni* populations. Comparatively speaking, the epidemic had a moderate impact on *E*. *troschelii*, *Henricia spp*. and subtidal *P*. *ochraceus* populations. No detectable impact was noted for *L*. *hexactis* and *Mediaster spp*. populations, and *D*. *imbricata* populations could even be increasing post SSWD outbreak.

Examples of other large-scale epidemics with a wide host range (affecting multiple different species) include chytridiomycosis, caused by *Batrachochytrium dendrobatidis*, in numerous amphibian species [42], rinderpest in African artiodactyls [43], and West Nile Virus in North American birds [44]. In all three examples, the overall impact has been devastating; however, like we show here, species-specific differences have been reported.

### Basin differences

No substantial differences were observed across the 5 basins in the Salish Sea and the Outer Coast. Abundance for *D*. *imbricata*, *P*. *helianthoides* and *P*. *brevispinus* varied by basin, but not convincingly enough to suggest that major differences in environmental factors either existed or played a role in disease outcome among basins. While low water temperature was associated with better short-term SSWD survival [14] and slower disease progression and mortality rate [10,13], in other regions SSWD signs increased with cooler temperatures [15]. While water temperature could have played a role in the onset and progression of disease by basin, we hypothesize this role would be most apparent during the initial phase of the epidemic. Our analysis was on a large time scale (by year) relative to disease progression (weeks); therefore, SSWD effects among basins could have ‘caught up’ and not been detectable in our analysis, even if initial progression was different. The role that food availability, crowding, and other environmental factors played in SSWD emergence and virulence is unknown.

### Future perspectives and trophic cascade

The SSWD epidemic has been characterized as the largest disease epidemic of a marine wildlife taxon based on the geographic range and number of species affected [9,10]. While the significant impacts to the dominant intertidal species *P*. *ochraceus* across a wide part of its range have been reported [10,14,15], we show that the impacts could be even greater to the subtidal star species *P*. *helianthoides*. Prior to the SSWD epidemic, this species was the most abundant subtidal asteroid in the Salish Sea [21,45]. Subtidal surveys analyzed here suggest *P*. *helianthoides* has been virtually extirpated from the Central Basin, Northern Straits basin and the Strait of Georgia basin, as well as on the Outer Coast. Anecdotal reports suggest the loss extends all the way to Alaska, as far north as Katchemak Bay. Recent diver observations from the Salish Sea confirm that adults are largely absent in the spring of 2016.

**Fig 10:**
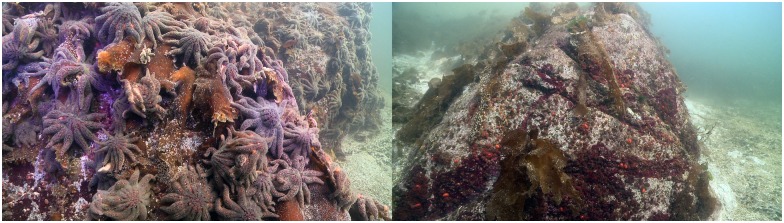
Comparative presence of *P*. *helianthoides—*October 2013—at a prominent rock near Vancouver, British Columbia, Canada (Strait of Georgia Basin). The pictures were taken within 3 weeks, prior to and after SSWD onset. Photo credit: Neil McDaniel, www.neilmcdaniel.com.

Wide host range pathogens can be particularly impactful because they can be maintained in more resistant species [46]. This could lead to the permanent exposure of more susceptible species, like *P*. *helianthoides*, to very low levels of infectious agent that could impede population recovery, and leave them prone to stochastic events. Moreover, disease has been shown to drive the extinction of wild populations [46]. This rapid and extensive loss warrants concern for the long-term survival of this species, which could require prompt management action (Fig 10).

The decline and near extirpation of a once highly abundant major subtidal predator such as *P*. *helianthoides* [47] has the potential to shift community composition [48]. In this case, due to the known consumption of urchins by *P*. *helianthoides* [47–50], the expected impact is an increase in *M*. *franciscanus* and *S*. *droebachiensis*, which could in turn decimate habitat-forming kelp beds and development of urchin barrens [48,51]. Trophic-level cascades consequent to SSWD have been suggested already. *Pisaster ochraceous* predation of the mussel *Mytilus californianus* decreased heavily along the Oregon State coast [15], while further north in Howe Sound (southern Strait of Georgia basin, British Columbia, Canada) devastating losses of *P*. *helianthoides* were accompanied by extreme increases in *S*. *droebachiensis* creating large areas devoid of kelp [36]. Our study was not designed to detect this kind of an impact, but the roving diver surveys do show *S*. *droebachiensis* increased in four out of six basins (Central Basin, Northern Straits, South Puget Sound and Strait of Georgia), with greatest increases in the Central Basin and South Puget Sound basins. *M*. *franciscanus* increased in all basins where they were present (Northern Straits basin, the Outer Coast and the Strait of Georgia basin), but markedly and sustained increases only occurred the in the Northern Straits basin. A potential reduction in *M*. *franciscanus* harvesting is not a likely explanation because similar urchin abundance patterns were seen when survey data was collected in *M*. *franciscanus* harvest closure sites in the Northern Straits basin [24].

Removing a keystone species can also have a diversifying effect on less dominant taxa and ultimately enhance species diversity by creating space for prey [52, 53]. It is too early to tell if less-impacted species like *D*. *imbricata*, *L*. *hexactis* and *Henricia spp*., will benefit from SSWD mortality in previously dominant asteroid species. As with other multi-species epidemics the long-term impact of SSWD on distribution and abundance of affected species will depend on species-specific differences in susceptibility and community-level changes associated altered predator abundance and prey availability.

## Supporting Information

S1 Appendix Species and basin specific ARIMA models and their estimated parameter values. (DOCX)

S2 Appendix Code for the multilevel Poisson regression. (DOCX)
