# Supplementary material for: Devastating Transboundary Impacts of Sea Star Wasting Disease on Subtidal Asteroids
Source: PLoS One. 2016 Oct 26;11(10):e0163190. doi: 10.1371/journal.pone.0163190 (PMC5082671; doi:10.1371/journal.pone.0163190)
Supplement: S1 Appendix — (DOCX) [file pone.0163190.s001.docx]

**S1 Appendix. Coefficients of the fitted ARIMA models for the abundance of sea star species in 5 Salish Sea basins and Washington State Outer Coast.**

|  |  |  |  | **Intercept** | | **AR** | | **MA** | |  |
| --- | --- | --- | --- | --- | --- | --- | --- | --- | --- | --- |
| **Species** | **Basin** | **Model** | **σ^2^** | Est. | S.E | Est. | S.E. | Est. | S.E | **AICc** |
| *D. imbricata* | Central Basin | ARIMA(0,1,1) | 0.123 |  |  |  |  | -0.191 | 0.529 | 12.250 |
|  | Hood Canal | ARMA(0,0) | 0.026 | 0.383 | 0.057 |  |  |  |  | -0.040 |
|  | Northern Straits | ARMA(0,0) | 0.019 | 0.595 | 0.049 |  |  |  |  | -2.540 |
|  | Outer Coast | ARMA(0,0) | 0.168 | 2.540 | 0.155 |  |  |  |  | 14.390 |
|  | South Puget Sound | ARMA(0,0) | 0.041 | 0.930 | 0.072 |  |  |  |  | 3.560 |
|  | Strait of Georgia | ARMA(0,0) | 0.099 | 0.926 | 0.112 |  |  |  |  | 10.630 |
| *P. brevispinus* | Central Basin | ARIMA(0,1,2) | 0.106 |  |  |  |  | 0.292 | 0.567 | 19.960 |
|  |  |  |  |  |  |  |  | -0.708 | 0.492 |  |
|  | Hood Canal | ARIMA(2,1,0) | 0.035 |  |  | 0.002 | 0.510 |  |  | 11.070 |
|  |  |  |  |  |  | 0.283 | 0.285 |  |  |  |
|  | Northern Straits | ARMA(0,0) | 0.035 | 0.197 | 0.067 |  |  |  |  | 10.000 |
|  | Outer Coast | ARMA(0,0) | 0.101 | 0.502 | 0.120 |  |  |  |  | 10.83 |
|  | South Puget Sound | ARIMA(1,1,0) | 0.084 |  |  | 0.0037 | 0.449 |  |  | 9.550 |
|  | Strait of Georgia | ARMA(0,0) | 0.042 | 0.430 | 0.072 |  |  |  |  | 3.720 |
| *P. helianthoides* | Central Basin | ARIMA(2,1,1) | 0.046 |  |  | 0.621 | 0.267 | -0.578 | 0.602 | 28.600 |
|  |  |  |  |  |  | -0.769 | 0.197 |  |  |  |
|  | Hood Canal | ARIMA(0,0,0) | 0.033 | 1.622 | 0.657 |  |  |  |  | 2.170 |
|  | Northern Straits | ARIMA(1,1,0) | 0.021 |  |  | 0.676 | 0.286 |  |  | 0.460 |
|  | Outer Coast | ARMA(0,0) | 0.186 | 2.225 | 0.163 |  |  |  |  | 15.070 |
|  | South Puget Sound | ARMA(0,0) | 0.163 | 1.716 | 0.143 |  |  |  |  | 14.590 |
|  | Strait of Georgia | ARIMA(0,0,0) | 0.170 | 1.151 | 0.146 |  |  |  |  | 14.910 |
| *M. franciscanus* | Central Basin | ARMA(0,0) | <0.001 | 0.000 | 0.001 |  |  |  |  | -78.570 |
|  | Hood Canal | ARMA(0,0) | <0.001 | 0.000 | 0.000 |  |  |  |  | -116.320 |
|  | Northern Straits | ARIMA(2,1,1) | 0.039 |  |  | -0.658 | 0.198 | -0.273 | 0.422 | 16.190 |
|  |  |  |  |  |  | -0.911 | 0.097 |  |  |  |
|  | Outer Coast | ARMA(0,0) | 0.062 | 0.201 | 0.094 |  |  |  |  | 7.350 |
|  | South Puget Sound | - | - |  |  | - | - |  |  | - |
|  | Strait of Georgia | ARMA(0,0) | 0.022 | 0.709 | 0.053 |  |  |  |  | -1.380 |
| *S. droebachiensis* | Central Basin | ARMA(0,0) | 0.056 |  |  |  |  |  |  | 6.080 |
|  | Hood Canal | ARMA(1,0) | 0.003 |  |  | 0.743 | 0.186 |  |  | -17.820 |
|  | Northern Straits | ARMA(0,0) | 0.033 | 0.331 | 0.064 |  |  |  |  | 1.840 |
|  | Outer Coast | ARMA(0,0) | 0.002 | 0.045 | 0.018 |  |  |  |  | -15.960 |
|  | South Puget Sound | ARMA(0,0) | 0.013 | 0.112 | 0.040 |  |  |  |  | -5.790 |
|  | Strait of Georgia | ARMA(0,0) | 0.036 | 0.286 | 0.067 |  |  |  |  | 10.840 |
