# Supplementary material for: Devastating Transboundary Impacts of Sea Star Wasting Disease on Subtidal Asteroids
Source: PLoS One. 2016 Oct 26;11(10):e0163190. doi: 10.1371/journal.pone.0163190 (PMC5082671; doi:10.1371/journal.pone.0163190)
Supplement: S2 Appendix — (DOCX) [file pone.0163190.s002.docx]

#Bayesian Multilevel Poisson Regression using R and cited packages.

#loading packages

library('R2jags')

library('plyr')

d<-read.csv('~/ /SubStars.m.csv') # loading data

d<-d[ order(d[,2], d[,4]),] #sorting

d<-d[d$Taxon!='Solaster.dawsoni',] # taking out this taxon as was absent in both years

d$Area<-d$TransNo* d$Tlength*d$Twidth # the area surveyed for each taxon count

d$year2<-ifelse(d$Year==2014,0,1) #categorizing the year variable

#### Setting data for jags ####

ntaxon<-length(Taxon)

nobs<-nrow(d)

taxon=id(list(d$Taxon), drop=T), ntaxon=ntaxon, nobs=nobs)

data <- list(count=d$count,year=d$year2, offset=log(d$Area),

####### Actual model for JAGS #####

modelstring="

model {for (i in 1:nobs){

count[i] ~ dpois (lambda[i])

log(lambda[i])<-offset[i] + a[taxon[i]] + b[taxon[i]]*year[i] + epsilon[i]

epsilon[i]~dnorm(0,tau.epsilon)

}

tau.epsilon <-pow(sigma.epsilon, -2)

sigma.epsilon~dunif(0,100)

for(j in 1:ntaxon){

a[j]<-B[j,1]

b[j]<-B[j,2]

B[j,1:2]~ dmnorm (B.hat[j,], Tau.B[,])

B.hat[j,1]<-mu.a

B.hat[j,2]<-mu.b

}

mu.a~dnorm(0, 0.0001)

mu.b~dnorm(0, 0.0001)

Tau.B[1:2, 1:2]<-inverse(Sigma.B[,])

Sigma.B[1,1]<-pow(sigma.a, 2)

sigma.a~dunif(0,100)

Sigma.B[2,2]<-pow(sigma.b, 2)

sigma.b~dunif(0,100)

Sigma.B[1,2]<-rho*sigma.a*sigma.b

Sigma.B[2,1]<-Sigma.B[1,2]

rho~dunif(-1,1)

}"

# Parameters of interest

param<-c('a', 'b' ,'epsilon','mu.a','mu.b','sigma.epsilon','sigma.a','sigma.b','rho')

# the random intercepts

# the random slopes

#the parameters allowing for overdisperion

#correlation matrix

#correlation matrix

#correlation matrix

# variance among intercepts

# variance among slopes

# correlation betwwen slopes and intercepts

#initial random values to start the MCMC

inits<-function (){

list(epsilon=rnorm(nobs), sigma.epsilon=runif(1), mu.a=rnorm(1), mu.b=rnorm(1), sigma.a=runif(1), sigma.b=runif(1), rho=runif(1))

}

# Running the model from R

model<-jags(data, inits, param, textConnection(modelstring), n.chains = 3,n.iter = 60000, n.thin = 5, n.burnin =10000)
